# Body Mass Index, Physical Activity, and Body Image in Adolescents

**DOI:** 10.3390/children9020202

**Published:** 2022-02-04

**Authors:** Marja H. Leppänen, Aku-Ville Lehtimäki, Eva Roos, Heli Viljakainen

**Affiliations:** 1 Folkhälsan Research Center, Topeliuksenkatu 20, 00250 Helsinki, Finland; aku-ville.lehtimaki@folkhalsan.fi (A.-V.L.); eva.roos@folkhalsan.fi (E.R.); heli.viljakainen@helsinki.fi (H.V.); 2 Faculty of Medicine, University of Helsinki, P.O. Box 20, 00014 Helsinki, Finland; 3 Department of Food Studies, Nutrition and Dietetics, Uppsala University, P.O. Box 256, SE-751 05 Uppsala, Sweden

**Keywords:** exercise, BMI, body size, children, cohort study

## Abstract

Body image dissatisfaction is a concern for adolescents’ mental and physical well-being, and the role of body mass index (BMI) and physical activity (PA) in it is still unclear. This study investigates the associations of BMI and PA with body image, separately for boys and girls, in a large sample of Finnish adolescents. We also examine the associations of BMI with body image in varying PA levels. A total of 10,496 adolescents (girls 52.6%) were included in the analyses. Body image was assessed using a pictorial tool, and categorized as wishing for a smaller body, being satisfied, and wishing for a bigger body. BMI (kg/m^2^) was categorized as thin, normal weight, and overweight/obese. Self-reported PA was divided into three similar-sized categories as low, moderate, and high PA levels. Adjusted ordinal regression analyses were conducted. Our results show that adolescents with thinness had higher odds of wishing for a bigger body compared to their normal-weight peers, while adolescents with overweight/obesity had smaller odds of wishing for a bigger body. Adolescents in low and middle PA levels had lower odds of wishing for a bigger body compared to adolescents in the high PA level. Yet, the PA level modified the associations between BMI and body image, especially in adolescents with thinness and more so in girls than in boys. These findings highlight the need to pay attention to healthy weight gain and PA in adolescents to support their body image satisfaction.

## 1. Introduction

Overweight is a complex condition with multiple contributory causes, including genetic, socioeconomic, environmental, and behavioral factors [1,2]. Over the past four decades, mean body mass index (BMI) and overweight have increased in children and adolescents in most countries worldwide [3]. In Finland, the prevalence of overweight/obesity was 28% in 7–12-year-old boys and 18% in girls in 2019 [4]. These numbers are alarming, since overweight is likely to track into adulthood [5]. Overweight has been associated with multiple physical and psychological consequences, such as an increased risk for cardiovascular diseases and decreased health-related quality of life [6]. Furthermore, a higher BMI has been linked with less favorable body image in young adults [7], which in turn, has been associated with poor psychological health [8,9].

Body image is a multidimensional construct referring to the psychological experience of embodiment, especially, but not exclusively, of one’s physical appearance [10]. It reflects how individuals think, feel, see, and act toward their bodies [11]. Body image dissatisfaction has been consistently rated as being among the foremost concerns for young people [12]. Although negative body perceptions have declined in many countries since 2014, every fourth of the adolescents perceive themselves as too fat, especially girls [13]. Body image has implications for one’s mental and physical health. For example, perceptions of poor body image are related to lower self-esteem [14], higher levels of depression and anxiety [15], and eating disorders [16]. Body image dissatisfaction may also increase the likelihood of obesity [17] as excessive preoccupation with appearance and pursuit of the ideal lean body can produce negative feelings affecting health behavior that will complicate weight management [18]. Nevertheless, concerns about body image have also been linked to reaching the idealized body, which in contemporary Western countries is a thin and a fit physique for females and a lean and muscular physique for males [19]. 

Physical activity (PA) has been suggested to be one way to achieve a body that resembles cultural expectations [20] and affects positively on body image [21]. A study in 1501 Spanish adolescents reported an inverse correlation between PA and body image, especially regarding moderate-to-vigorous PA [22]. Similar findings were observed in Poland in 3249 adolescents, and additionally, they reported stronger associations in boys compared to girls [23]. Yet, a study in 404 young adults in the USA found a positive correlation between body image and light PA, but not with moderate-to-vigorous PA [24]. BMI was controlled in all these studies indicating an independent role of PA in body image [22,23,24]. However, there is a lack of studies investigating how PA modifies associations of body image in different BMI categories in adolescents. As the role of weight status in body image may be different depending on the BMI category [21], increasing knowledge about how different levels of PA are related to these associations is important. Such information would help targeting supporting actions effectively on adolescents taking both BMI and PA into account in improving positive body image. Therefore, we aimed to examine associations of BMI and PA with body image in a large sample of 9 to 12-year-old Finnish adolescents. In addition, we examined whether the associations between body image and BMI are different in varying PA levels. The analyses were provided separately for boys and girls due to the previously reported sex differences [22].

## 2. Materials and Methods

### 2.1. Study Design and Participants

The present cross-sectional study utilized baseline data from the Finnish Health in Teens (Fin-HIT) cohort study conducted 2011–2014. Participants consisted of 11,407 adolescents at the age of 9 to 12 years from 44 municipalities, close to Finland’s largest cities: Helsinki, Turku, Espoo, Oulu, Jyväskylä, Tampere, and Kuopio. Briefly, the study was mainly carried out in a school setting without specific exclusion criteria [25]. Recruitment and data collection occurred in total in 496 schools and the response rate was 36%. Participating families were highly educated (55% had a university degree, *n* = 5834). An informed written consent was obtained from all adolescents and from one of their caregivers/parents. The study protocol was approved by the Coordinating Ethics Committee of the Hospital District of Helsinki and Uusimaa, Finland (Nr: 169/13/03/00/10). In this study, all adolescents with complete baseline information on body image, BMI, and PA were included (N = 10,496, girls 52.6%).

### 2.2. Body Image

A pictorial tool was used in evaluating body image, and it has been described in detail elsewhere [26]. In brief, the tool consisted of seven, numbered, sex-specific images illustrating body sizes ranging from very thin (number 1) to obese (number 7) [27]. Participants were asked: (1) which of the pictures most resembled your current body shape, and (2) which of the pictures most resembled the body shape you wanted. Based on their responses, the difference between the wanted and current body shape was calculated, and further, categorized into three groups: wishing for a smaller body, satisfied, and wishing for a bigger body. The group wishing for a smaller body included those who wished for a body shape that was smaller than the current one; the satisfied group included those who did not want a different body shape than their current one; and wishing for a larger body group included those who wished for a body shape that was larger than the current one. 

### 2.3. Anthropometry 

Weight (in kg) and height (in m) were measured by trained fieldworkers at schools with portable, daily-calibrated digital scales and a portable stadiometer. BMI was calculated as weight (kg)/height (m^2^), and it was further categorized as thin, normal weight, overweight, and obese according to Cole and Lobstein [28]. That reference system was chosen because it is concordant with the Finnish reference values [29,30]. The category of obese adolescents was small (2.6%); therefore, overweight and obese were combined.

### 2.4. Physical Activity 

PA was assessed using a question adapted from another Finnish school-based study [31]. The question was: “How many hours a week do you normally exercise or do sports during your free time? Include all the exercise you do in a club or team and any exercise by yourself, with family or friends. Do not count any exercise at school or on the way to school.” The response alternatives were ranging from (1) An hour a week or less to (10) Around ten hours a week. The question was targeted only to leisure-time PA, since it was intended to be easy to report. Moreover, leisure-time PA has been reported to cover the majority of the adolescents’ daily PA in Finland [32]. In the analyses, PA was recategorized into three similar-sized categories as the following: (1) around 5 h a week or less (low PA level), (2) around 6–8 h per week (moderate PA level), (3) around 9 h per week or more (high PA level). 

### 2.5. Other Background Data 

Sex and age (in years) of participants were confirmed by linkage with the National Population Information System at the Population Register Centre, Finland. Pubertal stage was self-assessed using the Tanner scale [33] and categorized into three groups: pre-pubertal, pubertal, and post-pubertal.

### 2.6. Statistical Analysis 

Descriptive information is presented as means and standard deviation (SD) or frequencies (%). Sex comparisons were made by t-test for continuous variables and Chi-square test for categorized variables, except Fisher’s exact test for pubertal stage due to the small sample size in the post-pubertal stage. Furthermore, Fisher’s exact test was used to test differences in BMI and PA categories across body image satisfaction (i.e., wishing for a smaller body, satisfied, wishing for a bigger body). An ordinal regression analysis was conducted first to examine the association of body image with (1) BMI and (2) PA, and secondly, to examine the association of body image with BMI stratified by PA level. Body image was ordered from the lowest to the highest as wishing for a smaller body, satisfied, and wishing for a bigger body. Since ordinal logistic regression was used, it was assumed that body image is actually a (latent) continuous variable, which has been only measured as a three-categorical variable; therefore, BMI and PA predict the changes in the latent continuous scale. Regarding BMI analyses, normal weight was used as a reference category, and regarding PA, the high PA level was used as a reference category. All models were adjusted for the child’s age and pubertal stage. The SPSS statistical software (version 26.0) was used for all statistical analyses, and a significance level was set to 5%. The figures were created using R package ggplot2. 

## 3. Results

Table 1 shows the characteristics of the 10,496 participating adolescents by sex. In general, boys had a lower pubertal stage, they were more satisfied with their body image, they were more often of normal weight, and had more PA compared to girls. 

The majority of boys with thinness (61%, 277 out of 455) or normal weight (71%, 2639 out of 3742) were satisfied with their body image, while 73% (568 out of 783) of boys with overweight wished for a smaller body (Table 2). Similarly, the majority of girls with thinness (60%, 422 out of 701) or normal weight (66%, 2624 out of 4001) were satisfied with their body, while 75% (609 out of 814) of girls with overweight wished for a smaller body, respectively. 

The distribution of body image satisfaction differed by PA level both in boys and girls (*p* < 0.001 for both) (Table 2). Boys with a high PA level were more satisfied with their body image (70%) than other boys with lower PA levels (57 and 59%). Correspondingly, boys with low PA wished for a smaller body more frequently (32%) than boys with higher PA levels (26%). Similar observations were noted in girls, with somewhat smaller differences.

### 3.1. Association of BMI and PA with Body Image

**Figure 1:**
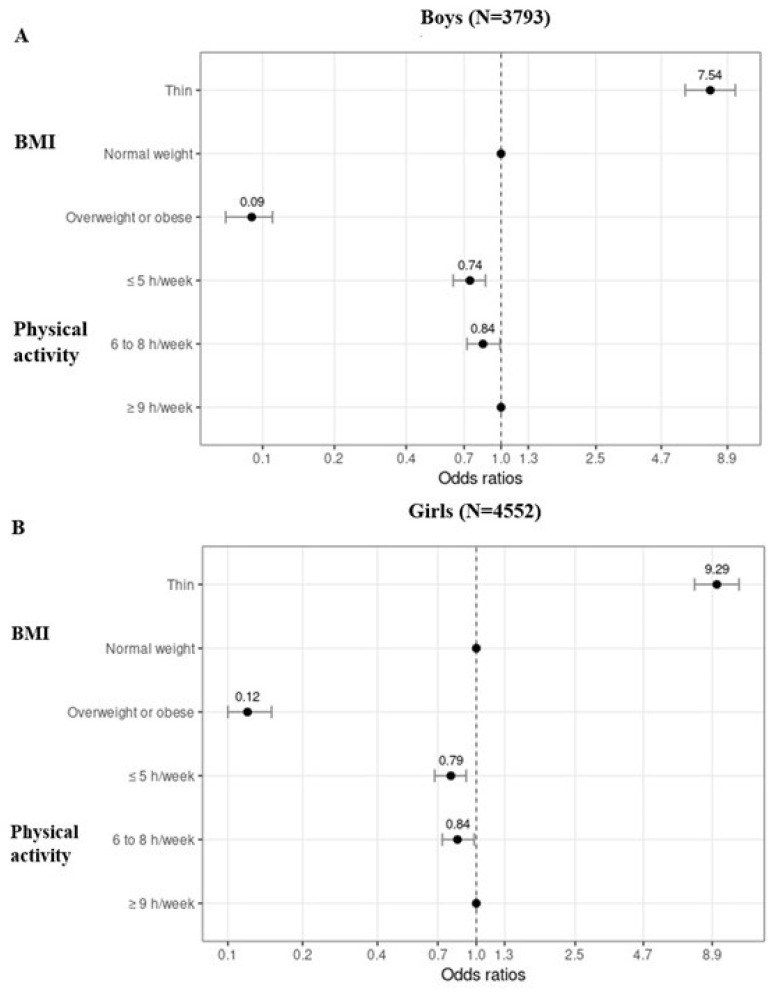
Odds ratios with their 95% confidence intervals showing the risk for wishing for a bigger body compared to being satisfied or wishing for a smaller body in different body mass index (BMI) and physical activity levels separately for boys (**A**) and girls (**B**). Normal weight and ≥9 h/week are reference groups.

Table 3 and Figure 1A,B show that boys and girls with thinness had higher odds of wishing for a bigger body than being satisfied or wishing for a smaller body compared to their counterparts with normal weight (both *p* < 0.001). In addition, boys and girls with overweight/obesity had smaller odds of wishing for a bigger body than being satisfied or wishing for a smaller body compared to their counterparts with normal weight (both *p* < 0.001). 

Boys and girls with low or moderate PA levels had smaller odds of wishing for a bigger body than being satisfied or wishing for a smaller body compared to their counterparts with a high PA level (all *p* < 0.031) (Table 3, Figure 1A,B).

### 3.2. Association of BMI with Body Image Stratified by PA Level

**Figure 2:**
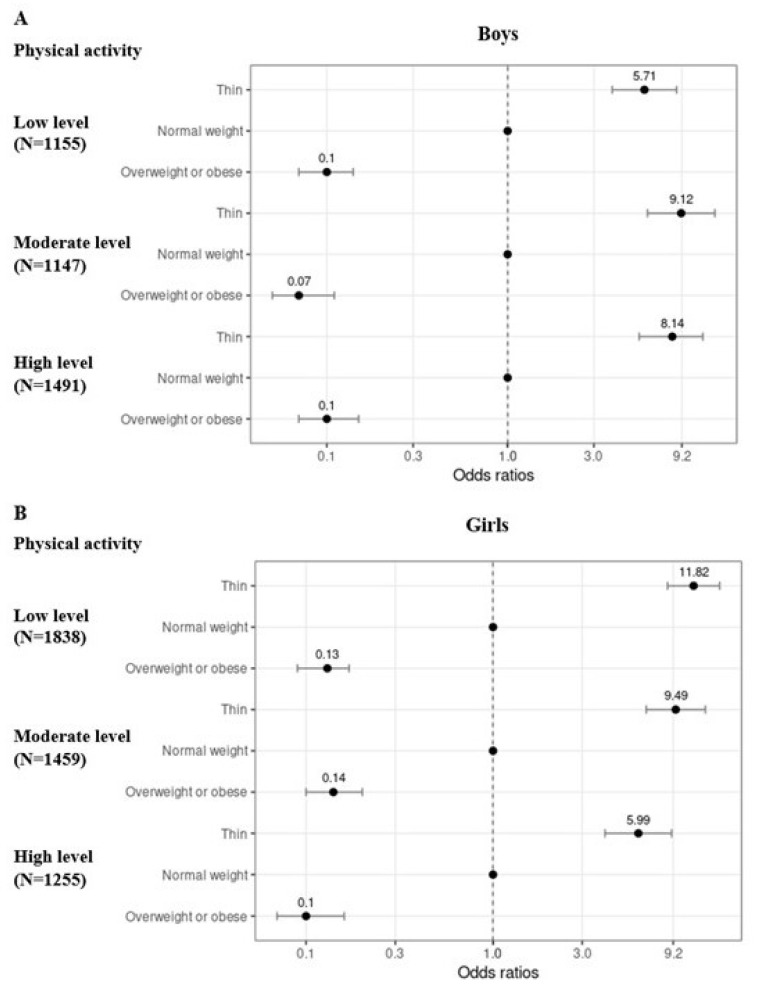
Odds ratios with their 95% confidence intervals from ordinal regression analyses showing the risk for wishing for a bigger body compared to being satisfied or wishing for a smaller body in different body mass index stratified by physical activity level separately for boys (**A**) and girls **(B**). Normal weight is a reference group. Low PA level indicates PA around 5 h a week or less, moderate PA level indicates PA around 6–8 h per week, and high PA level indicates PA around 9 h per week or more.

Table 4 and Figure 2A,B present that boys and girls with thinness had higher odds of wishing for a bigger body than being satisfied or wishing for a smaller body compared to their counterparts with normal weight in all PA levels (all *p* ≤ 0.001). Furthermore, boys and girls with overweight/obesity had smaller odds of wishing for a bigger body than being satisfied or wishing for a smaller body compared to their counterparts with normal weight in all PA levels (all *p* < 0.001).

The PA level modified the association between BMI and body image, especially in adolescents with thinness and more clearly in girls than in boys, while the same association was less affected by PA levels among adolescents with overweight. In girls, the lower the PA level, the higher the odds of wishing for a bigger body than being satisfied or wishing for a smaller body compared to their counterparts with normal weight. Whereas in boys with thinness, the modifying effect of PA was inconsistent, partly opposite to girls (Table 4, Figure 2A,B). 

## 4. Discussion

Our study shows that both BMI and PA are associated with body image in 9 to 12-year-old Finnish boys and girls. Although the associations were similar in boys and girls, girls had higher odds of wishing for a different body than their current body compared to boys. In addition, we observed that the PA level modified the associations between BMI and body image especially in adolescents with thinness, and more so in girls than in boys. 

In accordance with our results, the adolescents with thinness more commonly wished for a bigger body compared to their normal-weight peers. The associations were similar in boys and girls, but the magnitudes of the associations were stronger in girls compared to boys. This may indicate that girls are more worried about their thinness and wish for a bigger body compared to boys. Girls being more worried about their body image than boys has been reported before [34,35], for instance, due to higher social pressure. Nevertheless, when we took the PA level into account, the girls with thinness had the highest odds of wishing for a bigger body in a low PA level compared to higher PA levels. Furthermore, among girls with thinness, the odds of wishing for a bigger body decreased when the PA level increased. Yet, the trend was different in boys with thinness; the odds of wishing for a bigger body was the highest in the moderate PA level and the smallest in the low PA level. Girls with thinness were more satisfied with their body when they were physically more active, possibly due to increased lean body mass. In addition to that, a feeling that the body is improving through PA might be a reason for higher body image satisfaction [21]. In boys with thinness, however, the effect of the PA level was generally more consistent. Our findings are in line with a previous study proposing that PA protects body image satisfaction [35]. However, our study adds an important point of view concerning adolescents with thinness. Often wishing for a smaller body is typical in adolescents [36], but this seems to dependent on the BMI category as suggested before [34]. Thus, BMI should be taken into account when examining relationships with body image satisfaction, and further, planning strategies to improve it. In future studies, it would also be essential to clarify more carefully what kind of body adolescents with thinness are wishing for (e.g., more muscles or more shapes), and do they vary depending on what kind of PA they are engaging in (e.g., strength or endurance training). Furthermore, investigating associations between differences in body composition (such as body fat percentage or lean mass) and body image satisfaction would provide valuable information to health promotion work. 

Adolescents with overweight/obesity rarely wished for a bigger body compared to their normal-weight peers, and the magnitudes of the associations were rather consistent in boys and girls. This finding is consistent with the previous study in 234 Portuguese adolescents [35]. Furthermore, the associations were relatively similar in all PA levels suggesting that adolescents with overweight/obesity wished for a smaller body irrespective of their PA level. A previous study has concluded that moderate-to-vigorous PA has been positively related to body image independently of BMI [23]. Furthermore, in terms of wanting to lose weight, body image concerns have been lower in adolescents who participated in PA in which the performance does not depend on appearance or slimness (e.g., basketball or tennis), and in PA in which the performance is not based on appearance but on a lean physical body (e.g., swimming or athletics), as compared to those who participated in PA that aims to improve the physical appearance or for which physical appearance is important to success (e.g., fitness or gymnastics) or to those who were inactive [34]. However, methodological differences in assessing PA make it difficult to compare results between studies, and we, for instance, were not able to examine different PA intensities. Nevertheless, the adolescents in the current study were relatively young and the body image satisfaction may rely more on one’s outfit instead of behavior, for instance, due to the different stages of maturity. Therefore, it is possible that the body satisfaction increases “naturally”, if overweight declines with age [13]. Yet, our findings highlight the need to pay attention to healthy weight gain in young adolescents in order to support their body image satisfaction. 

We also observed that the adolescents in the low PA level had the lowest odds of wishing for a bigger body compared to adolescents in the high PA level. The same trend, although weaker, was seen in adolescents in the moderate PA level compared to adolescents in the high PA level. Since our PA questionnaires concerned only leisure-time, it is possible that the adolescents in the high PA level practiced sports where a bigger body is beneficial; therefore, they wished for it. Yet, our findings are in line with the previous studies in that promoting PA is important in order to support body image satisfaction in adolescents [22,37]. However, since most of the previous studies have been based on self-reported PA [37], the findings should be confirmed using objective measurements in assessing PA in order to be better able to clarify the role of PA. It is possible that different frequencies or intensities of the PA have varying effects on body image. It is also worth it to note that physical attractiveness in boys and athletic identity in both sexes have been positively associated with the intention to be physically active [38] which implies that the associations between PA and perceptions of one’s own body might be bidirectional.

The majority of the boys and girls were satisfied with their body image, and in addition, being a normal weight or having a high PA level were associated with higher body image satisfaction. Yet, four adolescents out of 10 were dissatisfied, and especially being overweight/obese was related to body image dissatisfaction. These findings are concerning, since being dissatisfied with one’s own body image may increase the risk for adverse consequences. Adolescents in the current study are in an age when their body shape is changing a lot. They are getting taller but also maturation changes their body and body shape. These changes may affect their body image satisfaction positively or negatively. Since a healthy relationship towards one’s own body is essential for physical and mental well-being [14,15,16], having a positive body image despite being overweight/obesity might prevent some of the long-term consequences of obesity. Although childhood obesity is a major health issue worldwide [3], attention is required also to adolescents with thinness or underweight to support their healthy growth. Encouraging adolescents to be physically active might be one tool to support body image satisfaction.

The strength of the present study is a large sample size, which made it possible to include adolescents across BMI categories (i.e., thin, normal weight, and overweight/obese). Furthermore, the large sample size allowed us to investigate boys and girls separately, which is crucial due to the sex differences in BMI, PA, and body image [22]. Relying on self-reported PA instead of objectively measured PA may be seen as a limitation. In addition, the pictorial tool we used in assessing body image does not allow us to ask about the content of the body, for instance, whether the adolescents wished for more muscles and/or more shapes. Therefore, future studies should address the body image more deeply. However, self-report tools are easy to administer and inexpensive, and thus, more feasible in large-scale studies. Finally, the cross-sectional study design limits the conclusion about causality between the observed associations.

## 5. Conclusions

This study shows that BMI and PA associate with body image satisfaction in a large sample of Finnish 9 to 12-year-old adolescents. Although the associations were similar in boys and girls, girls had higher odds of wishing for a different body than their current body compared to boys. In addition, we observed that the PA level modified the associations between BMI and body image more so in girls than in boys. Our findings highlight the need to pay attention to healthy weight gain in young adolescents in order to support their body image satisfaction, especially in girls. In addition, promoting PA would provide additional benefits. Since BMI influenced whether adolescents wished for a bigger or smaller body, BMI should be taken into account when examining body image in adolescents. Furthermore, in future studies, there is a need to clarify what kind of PA is needed to support body image satisfaction, and additionally, what kind of body image the adolescents are especially wishing for. Such knowledge would help in planning effective actions to improve healthy body image in adolescents.

## Figures and Tables

**Table 1:** Characteristics of participating adolescents (N = 10,496).

	Boys		Girls		*p*-Value ^a^
	N	Mean (SD)/%	N	Mean (SD)/%	
Age, years	4980	11.2 (0.84)	5516	11.1 (0.85)	0.34
Pubertal stage					<0.001
Pre-pubertal	1381	36.4	1477	32.4	
Pubertal	2381	62.8	3022	66.4	
Post-pubertal	31	0.8	53	1.2	
Body image satisfaction					<0.001
Wishing for smaller body	1358	27.3	1788	32.4	
Satisfied	3125	62.8	3245	58.8	
Wishing for bigger body	497	10.0	483	8.8	
Body mass index					<0.001
Thin	455	9.1	701	12.7	
Normal weight	3742	75.1	4001	72.5	
Overweight or obese	783	15.5	814	14.8	
Physical activity					<0.001
Low level	1599	32.1	2287	41.5	
Moderate level	1482	29.8	1718	31.1	
High level	1899	38.1	1511	27.4	

^a^ Sex comparisons were made by t-test for continuous variables and Chi-square test for categorized variables, except Fisher’s exact test for pubertal stage. Low PA level indicates PA around 5 h a week or less, moderate PA level indicates PA around 6–8 h per week, and high PA level indicates PA around 9 h per week or more.

**Table 2:** Body image satisfaction by body mass index and physical activity categories.

	Body Image Satisfaction			
	Wishing for Smaller Body	Satisfied	Wishing for Bigger Body	*p*-Value ^a^
	N (%)	N (%)	N (%)	
**Boys**	1358	3125	497	
Body mass index				<0.001
Thin	16 (3.5)	277 (60.9)	162 (35.6)	
Normal weight	773 (20.7)	2639 (70.5)	330 (8.8)	
Overweight or obese	569 (72.7)	209 (26.7)	5 (0.6)	
Physical activity				<0.001
Low level	507 (31.7)	916 (57.3)	176 (11.0)	
Moderate level	439 (29.6)	879 (59.3)	164 (11.1)	
High level	412 (21.7)	1330 (70.0)	157 (8.3)	
**Girls**	1788	3245	483	
Body mass index				<0.001
Thin	34 (4.9)	422 (60.2)	245 (35.0)	
Normal weight	1145 (28.6)	2624 (65.6)	232 (5.8)	
Overweight or obese	609 (74.8)	199 (24.4)	6 (0.7)	
Physical activity				<0.001
Low level	822 (35.9)	1240 (54.2)	225 (9.8)	
Moderate level	559 (32.5)	1022 (59.5)	137 (8.0)	
High level	407 (26.9)	983 (65.1)	121 (8.0)	

Values are frequencies (N) and percentages (%). ^a^ Body image satisfaction comparisons were completed using Fisher’s exact test. Low PA level indicates PA around 5 h a week or less, moderate PA level indicates PA around 6–8 h per week, and high PA level indicates PA around 9 h per week or more.

**Table 3:** Associations of body mass index and physical activity with body image satisfaction.

	Body Image Satisfaction	
	OR (95% CI)	*p*-Value
**Boys, N = 3793**		
Body mass index		
Thin	7.54 (5.92, 9.60)	<0.001
Normal weight	Ref.	
Overweight or obese	0.09 (0.07, 0.11)	<0.001
Physical activity		
Low level	0.74 (0.63, 0.86)	<0.001
Moderate level	0.84 (0.72, 0.99)	0.031
High level	Ref.	
**Girls, N = 4552**		
Body mass index		
Thin	9.29 (7.56, 11.41)	<0.001
Normal weight	Ref.	
Overweight or obese	0.12 (0.10, 0.15)	<0.001
Physical activity		
Low level	0.79 (0.68, 0.91)	0.001
Moderate level	0.84 (0.73, 0.98)	0.024
High level	Ref.	

Values are odds ratios (OR) with 95% confidence intervals (CI) with their *p*-values from ordinal regression analyses. Body image was ordered from the lowest to the highest as wishing for a smaller body, satisfied, wishing for a bigger body. All models were adjusted for child’s age and pubertal stage. Low PA level indicates PA around 5 h a week or less, moderate PA level indicates PA around 6–8 h per week, and high PA level indicates PA around 9 h per week or more.

**Table 4:** Associations of body mass index with body image satisfaction stratified by physical activity (PA) level.

		Body Image Satisfaction	
PA	BMI	OR (95% CI)	*p*-Value
**Boys**			
Low level (N = 1155)	Thin	5.71 (3.79, 8.61)	0.001
	Normal weight	Ref.	
	Overweight/obese	0.10 (0.07, 0.14)	<0.001
Moderate level (N = 1147)	Thin	9.12 (5.94, 14.01)	<0.001
	Normal weight	Ref.	
	Overweight/obese	0.07 (0.05, 0.11)	<0.001
High level (N = 1491)	Thin	8.14 (5.34, 12.01)	<0.001
	Normal weight	Ref.	
	Overweight/obese	0.10 (0.07, 0.15)	<0.001
**Girls**			
Low level (N = 1838)	Thin	11.82 (8.58, 16.29)	<0.001
	Normal weight	Ref.	
	Overweight/obese	0.13 (0.09, 0.17)	<0.001
Moderate level (N = 1459)	Thin	9.49 (6.60, 13.66)	<0.001
	Normal weight	Ref.	
	Overweight/obese	0.14 (0.10, 0.20)	<0.001
High level (N = 1255)	Thin	5.99 (3.97, 9.02)	<0.001
	Normal weight	Ref.	
	Overweight/obese	0.10 (0.07, 0.16)	<0.001

Values are odds ratios (OR) and 95% confidence intervals (CI) with their *p*-values from ordinal regression analyses. Body image was ordered from the lowest to the highest as wishing for a smaller body, satisfied, wishing for a bigger body. Regarding BMI analyses, normal weight was used as a reference category. All models were adjusted for child’s age and pubertal stage.

## Data Availability

The data are not publicly available due to research ethical reasons and because the owner of the data is the Samfundet Folkhälsan i svenska Finland r.f. and not the research group. However, the corresponding author can provide further information on the Fin-HIT study and the Fin-HIT data upon a reasonable request.
